# Validity of environmental audits using GigaPan^®^ and Google Earth Technology

**DOI:** 10.1186/s12942-018-0147-7

**Published:** 2018-07-06

**Authors:** Erica Twardzik, Cathy Antonakos, Ross Baiers, Tamara Dubowitz, Philippa Clarke, Natalie Colabianchi

**Affiliations:** 1 Environment and Policy Lab, School of Kinesiology, University of Michigan, 1402 Washington Heights, Ann Arbor, MI 48109-2013 USA; 2 Department of Epidemiology, School of Public Health, University of Michigan, Ann Arbor, MI USA; 3 RAND® Corporation, Pittsburgh, PA USA; 4 Institute for Social Research, University of Michigan, Ann Arbor, MI USA

**Keywords:** GigaPan^®^, Methodology, Environmental audit, Built environment

## Abstract

**Background:**

Health behaviors are shaped by the context in which people live. However, documenting environmental context has remained a challenge. More specifically, direct observation techniques require large investments in time and resources and auditing the environment through web-based platforms has limited stability in spatio-temporal imagery. This study examined the validity of a new methodology, using GigaPan^®^ imagery, where we took photos locally and, stitched them together using GigaPan^®^ technology, and quantified environmental attributes from the resulting panoramic photo. For comparison, we examined validity using Google Earth imagery.

**Methods:**

A total of 464 street segments were assessed using three methods: GigaPan^®^ audits, Google Earth audits, and direct observation audits. Thirty-seven different attributes were captured representing three broad constructs: land use, traffic and safety, and amenities. Sensitivity (i.e. the proportion of true positives) and specificity (i.e. the proportion of true negatives) were used to estimate the validity of GigaPan^®^ and Google Earth audits using direct observation audits as the gold standard.

**Results:**

Using GigaPan^®^, sensitivity was 80% or higher for 6 of 37 items and specificity was 80% or higher for 31 of 37 items. Using Google Earth, sensitivity was 80% or higher for 8 of 37 items and specificity was 80% or higher for 30 of 37 items. The validity of GigaPan^®^ and Google Earth was similar, with significant differences in sensitivity and specificity for 7 items and 2 items, respectively.

**Conclusion:**

GigaPan^®^ performed well, especially when identifying features absent from the environment. A major strength of the GigaPan^®^ technology is its ability to be implemented quickly in the field relative to direct observation. GigaPan^®^ is a method to consider as an alternative to direct observation when temporality is prioritized or Google Earth imagery is unavailable.

## Background

Built environment characteristics can encourage or constrain an individual’s ability to engage in healthy behaviors such as physical activity and dietary behaviors and have been identified as potential causal factors contributing to obesity and physical inactivity [1–6]. Built environment features include structures supportive of physical activity such as sidewalks, lighting, or buffers to separate pedestrians from motorized traffic. The built environment also includes features that may be barriers, such as the absence of curb cuts, litter, and highly trafficked roads. These environmental features can have a significant impact on individuals’ decisions to engage in physical activity behavior, making them a critical component when evaluating population health [7, 8]. Characterizing fine details of the built environment is a necessary component for: identifying specific features associated with health behaviors, examining current community environments for advocacy work, and documenting where resources are most needed within the local, state, or national level.

While research has documented the importance of place on health and health behaviors, reviews of current methodology used to document features within the built environment have concerning limitations that have been well established [9, 10]. The current gold standard for documenting features within the built environment is direct observation. Direct observation methods require people to walk or drive through an environment to document features around them [10]. However, this method requires significant time and financial resources, thus other methods with reduced cost or effort to collect these data are often used [11, 12]. Examples of these methods include self-report measures, archival data, and web-based audits [10]. Yet, each of these methods has notable limitations. Self-report measures are subject to same-source bias, where the outcome of interest may impact how people report or perceive surrounding environmental attributes [13]. Additionally, the administration of self-report questionnaires can be challenging due to low response rates from participants, and self-reported information has been shown to have low correlation with objective built environment measures [10, 14]. Archival data, such as from Geographic Information Systems (GIS), often lack information on specific exposures of interest, such as the quality of features within the built environment [10]. Moreover, considerable time and expense is required to create exposures from these data [10]. Furthermore, GIS data are restricted by the spatial scale and priorities of the organizations collecting the data, which may have different goals and purposes for the use of their archival data [15, 16]. Web-based audits, such as Google Earth, may have insufficient resolution for documenting important fine-grained attributes (e.g. presence of graffiti, advertising) and web-based imagery is not always available in remote areas of the world [17, 18]. In addition, the timing of web-based imagery is not controlled by the researcher and temporal change to environmental attributes are difficult to capture [19, 20]. Because we rely on these methods to provide evidence for community-based policies that are relevant to health, development in this area is critical.

**Fig. 1:**
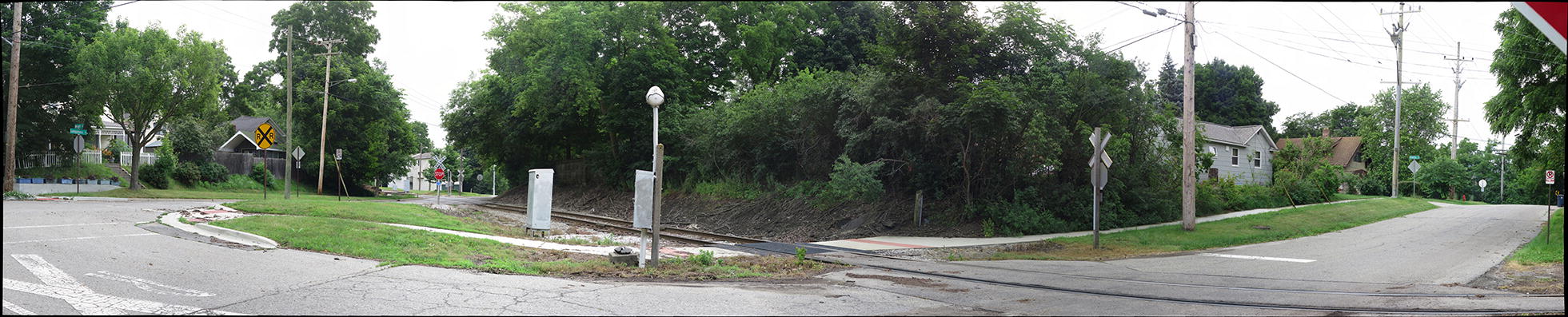
Example of a GigaPan^®^ panorama photo capturing a street segment not from this study using the GigaPan^®^ robotic camera mount (GigaPan^®^, Epic) along with an everyday camera (Canon^®^, PowerShot S120)

A potential solution is to document the environment using high-resolution imagery and document fine-grained environmental attributes. GigaPan^®^ is such a device that can capture high-resolution photos. GigaPan^®^ is a robotic camera-mounting device developed by Carnegie Mellon University and National Aeronautics and Space Administration (NASA) Ames Intelligent Robotics Group for use on NASA’s Mars Rovers. When mounted within the device, an everyday camera can automatically take thousands of photos using the device’s robotic arm. The resulting photos are combined into a single photo using GigaPan^®^ Stitch Software, producing an extremely high resolution and highly navigable panoramic picture that can cover large geographic spaces. The user can zoom into any part of the panoramic, stitched photo and see excellent detail. An example of a stitched GigaPan^®^ panorama is shown in Fig. 1 and additional examples can be explored at GigaPan.com. However, the potential use of GigaPan^®^ imagery in studies of the built environment has not been investigated and its performance relative to other methodology has not been characterized. This innovative technology may provide accurate representation of the built environment when other measures of characterizing the built environment are unavailable, too expensive, or the need for documentation during a specific time interval is a priority. This study was designed to estimate the validity of measures obtained via GigaPan^®^ in comparison with direct observation, the gold standard. As a comparable method, web-based Google Earth audits were completed for the same street segments and were examined for validity, also against direct observation.

## Methods

### Setting

To examine the validity of GigaPan^®^ methodology a total of 614 street segments were sampled from two communities in Pittsburgh, Pennsylvania. The study sample was comprised of street segments from 13 census tracts. Within this study, a street segment was defined as a segment extending from one street intersection to the next street intersection, and encompassing both sides of the street.

### Street segment observation form

To code built environment features, an audit tool developed by Bridging the Gap (BTG) called the ‘BTG Street Segment Observation Form’ was used [21]. The audit instrument was designed to assess key street-level features of the neighborhood environment that are thought to be related to physical activity behavior and active transport. The street segment observation form included 50 items to characterize the built and social environment, focusing on the following environmental constructs: land use, traffic and safety, and public amenities.

### Direct observation

Four trained neighborhood residents (i.e. community-based fieldworkers), trained as part of the staff, walked the length of each segment to complete an audit of the built environment for all street segments through direct observation. Fieldworkers recruited to complete direct observation of the built environment were formally trained in the use of the BTG Street Segment Observation Form through written and visual instruction. This included training both in the classroom and in the field on how to recognize the presence of features and how to rate the quality of the features, if applicable. Field Coordinators supplied staff with maps to indicate the street segments they were to observe and conducted field visits to ensure data collection quality. In pairs, field staff walked each street segment and completed the BTG Street Segment Observation Form. A community member hired as part of the research team oversaw data collection and reviewed 10% of the sample to identify and resolve any inconsistencies. All direct observation audits were completed from August 2015 to October 2015.

### GigaPan^®^

The GigaPan^®^ robotic camera mount (GigaPan^®^, Epic; $339.95) was used within the field along with an everyday camera (Canon^®^, PowerShot S120; $100.00). Four neighborhood residents were trained as staff (i.e. community-based fieldworkers), to collect GigaPan^®^ photos for this project. All community-based field workers who were taking GigaPan^®^ photos read the GigaPan^®^ manual, watched an instructional video, and completed practice photos in the field using the equipment. Each GigaPan^®^ photo was stitched together using the GigaPan^®^ Epic Stich software and labeled with a unique identifier corresponding to the street segment where it was taken. All GigaPan^®^ photos were taken from September 2015 to December 2015, stitched together on site, and then transported to a remote location for coding.

Remote coders were trained within a classroom setting where all coders read the manual and audit tool items were discussed in a group setting using written, visual, and verbal instructions. All coders were oriented to the street segment observation form and instructed on how to navigate GigaPan^®^ photos. Practice GigaPan^®^ photos were independently audited and then discussed within a group setting until there was consensus among all coders. Remote coders used a digital version of the direct observation street segment observation form for all practice audits. All coders were certified as reliable by achieving 80% reliability on a group of sample segments.

### Google Earth

Street segment audits were completed in Google Earth by replicating the process used in previous research [22]. A shapefile containing all the street segments was exported to a KMZ file to load into Google Earth. Each line segment was labeled with a unique identifier for the street. Once in Google Earth, coders were able to see the unique identifier provided by the KMZ file, and could easily navigate to the individual street segment and traverse along the segment of interest via Google Street View to identify features in the environment. The training for audits of Google Earth imagery was similar to the training for GigaPan^®^. Remote coders were trained within a classroom setting where all coders read through the manual and audit tool items were discussed in a group setting using written, visual, and verbal instructions. All coders were oriented to the digital street segment observation form and instructed on how to navigate street view within Google Earth. Practice Google Earth segments were identified for independent coding. All coders met with a reliability coder to discuss any disagreement in a group setting until there was consensus among all coders. Remote coders used a digital version of the direct observation street segment observation form for all practice audits and were provided five attempts to achieve 80% reliability before data collection. Google Earth imagery dates were collected throughout the coding process. Google collected and uploaded new imagery throughout the study period. Google Earth imagery dates ranged from July 2007 to September 2016.

The GigaPan^®^ or Google Earth imagery from 575 street segments were assigned to eight research staff for coding. Research staff were restricted to one audit for a unique street segment, however all research staff audited both GigaPan^®^ and Google Earth imagery. Research staff were restricted to auditing a unique street segment once to eliminate any environmental information the auditor could carryover from one method to another.

### Statistical analysis

To characterize the validity of each method against direct observation, we estimated sensitivity and specificity. A method’s sensitivity is defined as the proportion of “true positives” it detects (i.e. the proportion of present environmental features, as defined by direct observation, and also defined to be present by the alternative measurement method). A method’s specificity is defined as the proportion of “true negatives” it detects (i.e., the proportion of absent environmental features, as defined by direct observation, and also defined to be absent by the alternative measurement method). To evaluate the relative performance of the alternative measurement methods, we compared confidence intervals. Non-overlapping confidence intervals provide a conservative estimate of a statistically significant difference between measures [23, 24].

To calculate sensitivity and specificity all variables were recoded as dichotomous, with 1 indicating the presence of a feature and 0 indicating absence. For measures including ‘neither’, ‘one’, or ‘both sides’ of the street as response categories, (e.g. sidewalk, detached housing) data were recoded to represent ‘neither’ in comparison to ‘one or more sides of the street’. For one ordinal item, the number of traffic lanes, the recoded indicator represents ‘zero and one lane’ in comparison to ‘two or more lanes’ of traffic. Variables previously found to have low reliability (Kappa < 0.20) were excluded from validity analysis. Sensitivity and specificity was calculated using the full analytic sample. In addition, in an effort to minimize bias, sensitivity and specificity were calculated on a second, more robust subsample, which excluded segments with any documented problems. All analyses were performed using STATA, version 14.2 (StataCorp LP, College Station, Texas).

## Results

**Fig. 2:**
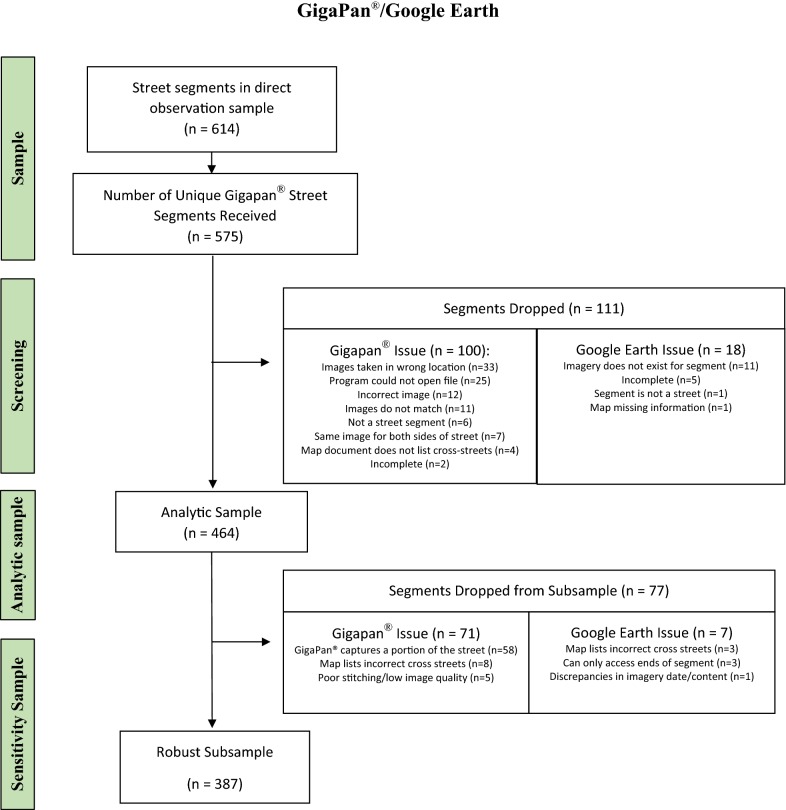
Flowchart depicting street segment sampling, along with detailed rational for exclusion. The analytic sample was presented and evaluated for validity in the current study

A total of 464 street segments were included in our analytic sample (Fig. 2). Of the 614 street segments within the study sample, a total of 575 were documented using a GigaPan^®^ photo. Of the 575 unique street segments, 100 were excluded from the analysis due to GigaPan^®^ imagery issues (e.g. images taken in wrong location, segment is not a street). Additionally, 18 street segments were excluded from the analysis due to Google Earth imagery issues (e.g. images taken in wrong location, segment is not a street). A total of 7 street segments had documented problems for both GigaPan^®^ and Google Earth, and when accounting for that overlap, 111 total segments were excluded from the analysis (Fig. 2).

A total of 37 variables were characterized into three broad constructs: land use, traffic and safety, and public amenities.

### Land use

As shown in Table 1, when using direct observation audits as the gold standard, GigaPan^®^ and Google Earth audits both correctly identified the presence of stand-alone detached housing at least 80% of the time. Google Earth audits were significantly better than GigaPan^®^ audits at identifying the presence of attached housing, institutional land use, total number of churches, bars on windows, and broken or boarded windows. GigaPan^®^ audits were able to correctly identify the absence of all 13 land use items at least 80% of the time. Google Earth audits correctly identified the absence of 12 of 13 land use variables with 80% or higher specificity. GigaPan^®^ audits were significantly better than Google Earth audits at identifying the absence of bars on windows, and broken or boarded windows in this sample.

**Table 1:** Validity of GigaPan^®^ and Google Earth technology in comparison to direct observation for all land use categories for street segments in Pittsburgh, PA

Variable	N	Prevalence^a^	GigaPan^®^ versus direct observation				Google Earth versus direct observation			
			Sensitivity	95% CI	Specificity	95% CI	Sensitivity	95% CI	Specificity	95% CI
Housing detached	459	54.0	80.2	(74.7, 85.0)	81.0	(75.1, 86.1)	89.5	(85.0, 93.0)	79.6	(73.5, 84.8)
Housing attached	461	32.5	*35.3*	(27.7, 43.5)	92.3	(88.7, 95.0)	*52.7*	(44.4, 60.9)	87.5	(83.3, 90.9)
Institutional	462	19.7	*44.0*	(33.6, 54.8)	98.9	(97.3, 99.7)	*71.4*	(61.0, 80.4)	96.2	(93.7, 97.9)
Service	463	17.5	39.5	(28.8, 51.0)	97.9	(95.9, 99.1)	39.5	(28.8, 51.0)	95.8	(93.3, 97.6)
Public/civic	459	6.3	0 06.9	(00.8, 22.8)	98.8	(97.3, 99.6)	37.9	(20.7, 57.7)	98.6	(97.0, 99.5)
Restaurants total	464	3.9	50.0	(26.0, 74.0)	99.6	(98.4, 99.9)	55.6	(30.8, 78.5)	98.0	(96.2, 99.1)
Churches total	464	9.3	*30.2*	(17.2, 46.1)	97.1	(95.1, 98.5)	*67.4*	(51.5, 80.9)	97.1	(95.1, 98.5)
Car repair shop	464	1.7	50.0	(15.7, 84.3)	98.7	(97.2, 99.5)	37.5	(08.5, 75.5)	97.6	(95.7, 98.8)
Bars total	464	1.9	11.1	(00.3, 48.2)	99.6	(98.4, 99.9)	22.2	(02.8, 60.0)	98.7	(97.2, 99.5)
Retail	461	9.3	34.9	(21.0, 50.9)	94.7	(92.1, 96.7)	60.5	(44.4, 75.0)	94.0	(91.3, 96.1)
Standalone court	460	1.5	14.3	(00.4, 57.9)	99.3	(98.1, 99.9)	42.9	(09.9, 81.6)	100	(99.2, 100)
Bars on windows	458	33.6	*22.1*	(15.8, 29.5)	*97.7*	(95.3, 99.1)	*51.3*	(43.1, 59.4)	*85.5*	(81.1, 89.3)
Broken/Board^b^ windows	462	34.0	*30.6*	(23.5, 38.4)	*94.8*	(91.6, 97.0)	*59.2*	(51.1, 67.0)	*84.9*	(80.4, 88.7)

Non-overlapping 95% confidence intervals for GigaPan^®^ and Google Earth sensitivity/specificity are in italic

*CI* confidence interval

^a^Percentage of segments with attribute, as determined by direct observation audit

^b^Broken or boarded up windows

### Traffic and safety

Of the sixteen traffic and safety variables, GigaPan^®^ audits correctly identified the presence of features in the environment with 80% sensitivity or higher for five of the sixteen variables (Table 2). Google Earth audits performed similarly, by correctly identifying traffic and safety features in the environment at least 80% of the time for seven of the sixteen variables. Google Earth audits were significantly better than GigaPan^®^ audits at identifying the presence of a marked crosswalk. GigaPan^®^ audits correctly identified the absence of ten of the sixteen traffic and safety features at least 80% of the time. Google Earth audits correctly identified the absence of twelve of the sixteen traffic and safety features at least 80% of the time. There were no significant differences between GigaPan^®^ and Google Earth audits in correctly identifying the absence of traffic and safety features in this sample.

**Table 2:** Validity of GigaPan^®^ and Google Earth technology in comparison to direct observation for all traffic and safety categories for street segments in Pittsburgh, PA

Variable	N	Prevalence^a^	Gigapan^®^ versus direct observation				Google Earth versus direct observation			
			Sensitivity	95% CI	Specificity	95% CI	Sensitivity	95% CI	Specificity	95% CI
Number of lanes of traffic^b^	464	81.3	83.0	(78.8, 86.7)	56.3	(45.3, 66.9)	85.9	(82.0, 89.3)	57.5	(46.4, 68.0)
Street type	462	05.8	59.3	(38.8, 77.6)	98.9	(97.3, 99.6)	66.7	(46.0, 83.5)	98.4	(96.7, 99.4)
Special speed limit	457	02.6	16.7	(02.1, 48.4)	99.6	(98.4, 99.9)	41.7	(15.2, 72.3)	98.2	(96.5, 99.2)
Curb	463	80.1	95.1	(92.4, 97.1)	79.3	(69.6, 87.1)	97.0	(94.8, 98.5)	82.6	(73.3, 89.7)
Street or sidewalk lighting	459	95.6	90.9	(87.8, 93.4)	65.0	(40.8, 84.6)	88.2	(84.8, 91.0)	75.0	(50.9, 91.3)
Sidewalk	457	80.1	97.0	(94.7, 98.5)	82.4	(73.0, 89.6)	98.1	(96.1, 99.2)	82.4	(73.0, 89.6)
Street and sidewalk buffer	350	39.1	70.1	(61.7, 77.6)	79.8	(73.8, 85.0)	78.1	(70.2, 84.7)	72.3	(65.8, 78.2)
Continuous sidewalk	351	96.6	90.9	(87.3, 93.7)	00.0	(00.0, 26.5)	87.6	(83.6, 90.9)	33.3	(9.9, 65.1)
Continuous sidewalk both ends	350	96.6	69.8	(64.6, 74.7)	75.0	(42.8, 94.5)	78.4	(73.6, 82.7)	83.3	(51.6, 97.9)
Traffic light	462	18.2	79.8	(69.6, 87.7)	97.4	(95.2, 98.7)	92.9	(85.1, 97.3)	98.4	(96.6, 99.4)
Ped. signal at traffic light	462	06.5	53.3	(34.3, 71.7)	97.7	(95.8, 98.9)	73.3	(54.1, 87.7)	98.1	(96.4, 99.2)
Stop sign	459	39.4	79.0	(72.3, 84.7)	91.7	(87.8, 94.7)	86.2	(80.3, 90.9)	92.1	(88.3, 95.0)
Children/special pop sign	461	05.0	13.0	(02.8, 33.6)	98.9	(97.4, 99.6)	34.8	(16.4, 57.3)	97.9	(96.1, 99.1)
Marked bike lane	462	03.7	52.9	(27.8, 77.0)	99.6	(98.4, 99.9)	47.1	(23.0, 72.2)	99.8	(98.8, 100)
Marked crosswalk	459	32.7	*56.0*	(47.7, 64.1)	92.9	(89.4, 95.5)	*72.0*	(64.1, 79.0)	93.9	(90.6, 96.3)
Median with traffic island	463	02.4	63.6	(30.8, 89.1)	100.0	(99.2, 100)	63.6	(30.8, 89.1)	100	(99.2, 100)

Non-overlapping 95% confidence intervals for GigaPan^®^ and Google Earth sensitivity/specificity are in italic

*CI* confidence interval, *Ped.* pedestrian, *Pop* population

^a^Percentage of segments with attribute, as determined by direct observation audit

^b^Lanes of vehicular traffic was categorized into one or fewer traffic lanes versus more than one traffic lane

### Public amenities

As shown in Table 3, GigaPan^®^ and Google Earth did not have any variables within public amenities that it correctly identified as present 80% of the time or more. Google Earth audits were significantly better at identifying the presence of a garden, a flower bed, or planters within this sample, although the sensitivity for Google Earth was below 80% (i.e., 65.3). GigaPan^®^ audits correctly identified the absence of all eight amenity and disorder items at least 80% of the time, while Google Earth audits correctly identified the absence of six of the eight amenity and disorder variables at 80% specificity. There were no significant differences between GigaPan^®^ and Google Earth audits in correctly identifying the absence of public amenities in this sample.

**Table 3:** Validity of GigaPan^®^ and Google Earth technology in comparison to direct observation for all public amenity categories for street segments in Pittsburgh, PA

Variable	N	Prevalence^a^	GigaPan^®^ versus direct observation				Google Earth versus direct observation			
			Sensitivity	95% CI	Specificity	95% CI	Sensitivity	95% CI	Specificity	95% CI
Garden, flowers, or planter	461	15.6	*34.7*	(23.9, 46.9)	80.5	(76.2, 84.3)	*65.3*	(53.1, 76.1)	73.3	(68.6, 77.6)
Public trash can	460	14.8	47.1	(34.8, 59.6)	96.7	(94.4, 98.2)	67.6	(55.2, 78.5)	94.1	(91.3, 96.2)
Bus stop	459	22.0	59.4	(49.2, 69.1)	96.9	(94.6, 98.5)	70.3	(60.4, 79)	95.8	(93.2, 97.6)
Bench/shelter at transit	462	2.8	61.5	(31.6, 86.1)	98.9	(97.4, 99.6)	61.5	(31.6, 86.1)	99.3	(98.1, 99.9)
Benches or other seating	460	1.3	0.0	(0, 45.9)	99.6	(98.4, 99.9)	16.7	(0.4, 64.1)	98.7	(97.1, 99.5)
Bicycle Parking	460	0.7	0.0	(0, 70.8)	99.3	(98.1, 99.9)	33.3	(0.8, 90.6)	98.9	(97.5, 99.6)
Amount street trees	463	32.8	61.8	(53.6, 69.6)	83.3	(78.7, 87.3)	69.7	(61.8, 76.9)	79.7	(74.8, 84.1)
Trees shading sidewalk	461	25.2	41.4	(32.3, 50.9)	89.0	(85.2, 92.1)	56.0	(46.5, 65.2)	89.3	(85.5, 92.3)

Non-overlapping 95% confidence intervals for GigaPan^®^ and Google Earth sensitivity/specificity are in italic

*CI* confidence interval

^a^Percentage of segments with attribute, as determined by direct observation audit

### Robust subsample

Within the robust subsample, 77 street segments had documented issues that were excluded (Fig. 2). After computing sensitivity and specificity within this subsample, parameter estimates were similar to estimates obtained in the analytic sample. Therefore, only results from the analytic sample are presented.

## Discussion

This study evaluated the validity of measures obtained using GigaPan^®^ imagery by comparing them to the same measures obtained via direct observation (gold standard) and web-based Google Earth audits (less time and cost). Among items measured using GigaPan^®^, 16.2% exhibited high sensitivity (sensitivity > 80%) and 83.8% exhibited high specificity (specificity > 80%). Google Earth performed similarly to GigaPan^®^ with 21.6% exhibiting high sensitivity and 81.1% exhibiting high specificity. Given the results of this study, GigaPan^®^ was shown to be a reasonable method for characterizing the built environment. A major difference in validity between the GigaPan^®^ and Google Earth methods is that GigaPan^®^ performed better overall for specificity (i.e. when an attribute was coded as absent via direct observation, GigaPan^®^ audits identified the absence of the attribute correctly). On the other hand, the Google Earth method provided better overall sensitivity of environmental features (i.e. when an attribute was coded as present via direct observation, Google Earth audits identified the attribute correctly).

The GigaPan^®^ methodology had a number of strengths. First, a major strength of the GigaPan^®^ technology is its ability to be implemented quickly in the field. Temporal change to environmental attributes may be difficult to capture using Google Earth because researchers cannot control the timing of imagery [25]. GigaPan^®^ serves as a potential solution to capture temporal changes in the environment, allowing researchers to study behavior changes in response to an environmental change. Second, GigaPan^®^ photographs provided fine-grained detail of entire street segments for remote coders to evaluate. The panoramic photos were able to be digitally stored in one central location. In our study, one panoramic photo and all associated images used to create that file was around 30–50 mega-bytes when the files are utilized within the GigaPan^®^ format, however when exported to a tif file as a single panoramic the size increases substantially. Third, measuring characteristics of the built environment has remained a challenge in both national and international settings. GigaPan^®^ may serve as a potential solution to unique challenges faced using virtual audits (e.g. Google Earth), such as limited imagery in international or rural environments [19, 26]. Previous research has demonstrated the feasibility of training international citizens to use innovated technology that captures information about the neighborhood environment [27, 28]. Utilizing citizen scientists and GigaPan^®^, photos could be captured and audited for features of the environment. Fourth, with GigaPan^®^, data collectors, who take the photos, can be trained remotely and not incur travel expenses. Using laypersons to obtain the photos could result in cost savings in multi-regional, national, or international studies. The cost of materials needed to obtain GigaPan^®^ panoramic photos amounted to less than $500 ($339.95 for GigaPan^®^ Epic, $100.00 for a Canon camera, $23.00 64 GB SD card, $4.00 for AA batteries, $30.00 for a tripod), assuming availability of a computer. Additionally, there has been increased availability of open source technologies that mimic the capabilities of GigaPan^®^. Technologies such as Hugin, which provide users the capability of combining overlapping images to allow for fine grain detail within a panorama [29]. Therefore, this technology may have external generalizability beyond the use of a GigaPan^®^ device. However, given the comparability of the results, between GigaPan^®^ and GSV, if imagery is available and time appropriate, GSV is free and requires no time to collect.

Using GigaPan^®^ to document built environment attributes also had notable limitations. GigaPan^®^ is limited by its single vantage point to document the environment. Since the panorama was taken from a single vantage point, coders had difficulty viewing features around obstructions (e.g. cars, signs). Furthermore, because the length of the segment is highly variable the GigaPan^®^ photo was clearer for a short segment and provided additional challenges for a long segment. Multiple photos along the segment can provide details for longer segments, however this introduces additional administrative challenges for documenting and completing an audit. There were also limitations of this study as a whole. Direct observation audits were not obtained at the same time that GigaPan^®^ or Google Earth imagery was taken, which could have limited the validity of both methods of environmental audits. The imagery obtained by community members using GigaPan^®^ was captured from 0 to 145 days after direct observation whereas Google Earth imagery was taken anywhere from 0 to 3003 days before or after direct observation. Additionally, while we had an original sample of 575 street segments, our analysis sample was reduced to 464 street segments due to issues with GigaPan^®^ and Google Earth imagery (Fig. 2). The most commonly reported problems included GigaPan^®^ capturing a fraction of the street segment, missing imagery, and capturing a segment that was not a street.

## Conclusion

Using the GigaPan^®^ methodology provides objectively measured micro-scale environmental features, which is a limitation of previous studies using GIS to characterize the built environment [30]. GigaPan^®^ audits obtained comparable results as Google Earth audits when capturing the built environment, and serves as a potential alternative to direct observation methods. Although the results were comparable the time involved in getting images to audit was not comparable to Google Earth. Google Earth is readily available while GigaPan^®^ photos need to be taken. Within this project we were able to implement GigaPan^®^ technology quickly in the field. GigaPan^®^ could be used in the future to capture temporal changes to environmental attributes. GigaPan^®^ provides an accurate representation of the built environment when other measures of characterizing the built environment are unavailable, too expensive, or timing of imagery is prioritized. Future research should examine the association between built environment attributes characterized using GigaPan^®^ and health behaviors (e.g. physical activity) or health outcomes (e.g. obesity) to determine its predictive validity. This method could be of great value to the field, particularly in remote areas where web-based imagery is not currently available.
